# Reduced expression of the Ion channel CFTR contributes to airspace enlargement as a consequence of aging and in response to cigarette smoke in mice

**DOI:** 10.1186/s12931-019-1170-3

**Published:** 2019-09-02

**Authors:** Jack H. Wellmerling, Sheng-Wei Chang, Eunsoo Kim, Wissam H. Osman, Prosper N. Boyaka, Michael T. Borchers, Estelle Cormet-Boyaka

**Affiliations:** 1 Department of Veterinary Biosciences, College of Veterinary Medicine, The Ohio State University, Columbus, OH USA; 2 Department of Internal Medicine, College of Medicine, University of Cincinnati, Cincinnati, OH USA

**Keywords:** CFTR, Emphysema, Smoking, Aging

## Abstract

Chronic Obstructive Pulmonary Disease (COPD) is a complex disease resulting in respiratory failure and represents the third leading cause of global death. The two classical phenotypes of COPD are chronic bronchitis and emphysema. Owing to similarities between chronic bronchitis and the autosomal-recessive disease Cystic Fibrosis (CF), a significant body of research addresses the hypothesis that dysfunctional CF Transmembrane Conductance Regulator (CFTR) is implicated in the pathogenesis of COPD. Much less attention has been given to emphysema in this context, despite similarities between the two diseases. These include early-onset cellular senescence, similar comorbidities, and the finding that CF patients develop emphysema as they age. To determine a potential role for CFTR dysfunction in the development of emphysema, *Cftr*^+/+^ (Wild-type; WT), *Cftr*^+/−^ (heterozygous), and *Cftr*^−/−^ (knock-out; KO) mice were aged or exposed to cigarette smoke and analyzed for airspace enlargement. Aged knockout mice demonstrated increased alveolar size compared to age-matched wild-type and heterozygous mice. Furthermore, both heterozygous and knockout mice developed enlarged alveoli compared to their wild-type counterparts following chronic smoke exposure. Taken into consideration with previous findings that cigarette smoke leads to reduced CFTR function, our findings suggest that decreased CFTR expression sensitizes the lung to the effects of cigarette smoke. These findings may caution normally asymptomatic CF carriers against exposure to cigarette smoke; as well as highlight emphysema as a future challenge for CF patients as they continue to live longer. More broadly, our data, along with clinical findings, may implicate CFTR dysfunction in a pathology resembling accelerated aging.

## Introduction

Chronic Obstructive Pulmonary Disease (COPD) is the third leading cause of death globally [1]. COPD is primarily caused by tobacco smoking, however other factors such as air pollution, individual genetics, and aging are also thought to play a role. COPD has traditionally been associated with two main phenotypes- chronic bronchitis and emphysema. Chronic bronchitis is accompanied by chronic airway inflammation and encompasses mucus hypersecretion, smooth muscle constriction, and small-airway fibrosis. Emphysema, also associated with inflammation, is characterized by airspace enlargement and tissue destruction in the lung parenchyma which results in alveolar distension and contributes to respiratory decline.

Many mechanistic similarities between aging and the development of COPD have been drawn and reviewed extensively, in which COPD can be considered an “accelerated aging” disorder [2]. While smoking is the primary cause of COPD, the finding that most smokers do not develop the disease has led to considerable interest in genetic factors that may predispose an individual to the effects of tobacco smoke. In accordance with the accelerated aging hypothesis, mutations in the genes encoding Sirtuin 2 [3] and Telomerase Reverse Transcriptase [4] have been found to be associated with COPD. Another potentially interesting gene in the context of aging and COPD is *CFTR*, encoding the Cystic Fibrosis Transmembrane conductance Regulator (CFTR).

CFTR is an anion channel involved in airway hydration and mucociliary clearance most commonly studied in the context of Cystic Fibrosis (CF). CF is a life-limiting autosomal-recessive disease in which severely reduced CFTR function increases patients’ susceptibility to lung infection and excessive inflammation, resulting in lung damage and ultimately respiratory failure [5]. CF patients also experience several symptoms often associated with aging, including diabetes and bone disease [6]; common comorbidities encountered in COPD [7]. An interesting clinical observation from CF patients undergoing lung transplantation is that they commonly develop emphysema, which becomes more severe with age [8]. Mechanistically, it is not clear whether this is due to the excessive airway inflammation associated with CF, or an alternative phenomenon. Interestingly, several markers of cellular senescence have been found to be increased in CF airways [9]. Additionally, skin fibroblasts from CF patients have been shown to senesce more readily than those from healthy controls [10].

A growing body of research supports the idea that COPD is a disease of acquired CFTR dysfunction, particularly in the context of the chronic bronchitis phenotype [11]. We have previously shown that CFTR expression is reduced in the bronchial epithelium of patients with severe COPD [12], and identified a mechanism by which cigarette smoke exposure leads to CFTR degradation [13]. Considering the similarities between CF and COPD, we hypothesized that disrupting CFTR expression may promote development of emphysema as a consequence of aging or exposure to tobacco smoke independent of CF airway disease. Mice do not develop the spontaneous infection and inflammation associated with CF airway disease [14, 15], providing a convenient model to address this question. To determine a potential role for CFTR expression levels in developing emphysema, the effects of aging and cigarette smoke exposure on *Cftr*^+/+^ (WT), *Cftr*^+/−^ (Het), and *Cftr*^−/−^ (KO) mice were examined.

## Methods

To determine a possible role for CFTR expression in emphysema-like changes, mice lacking CFTR (KO) or heterozygous (Het) for *Cftr*^*tm1Unc*^ [stop codon in the murine *cftr* gene (S489X)] and homozygous for Tg(FABP-CFTR) [fatty acid-binding protein (FABP)-CFTR]; or wild-type (WT) littermates (*Cftr*^*tm1Unc*^ FABP-hCFTR-CFTR bitransgenic mice from Jackson Laboratory, Bar Harbor, ME) were aged 14–20 months under standard pathogen-free housing conditions. Mice were considered “aged” at 14 months, because this age corresponds to the upper limit of a “middle-aged” mouse (https://www.jax.org/research-and-faculty/research-labs/the-harrison-lab/gerontology/life-span-as-a-biomarker). KO mice express human WT CFTR in the intestine (https://www.jax.org/strain/002364) and thus were able to be fed only standard mouse chow [16]. Seven-nine mice of each genotype were aged. Following euthanasia via CO_2_ and cervical dislocation, lungs were inflated with 10% neutral-buffered formalin at a pressure of 20 cmH_2_O and fixed for 24 h, sectioned, and stained with hematoxylin and eosin for morphometric analysis [17]. Alveolar airspace enlargement was quantified by calculating the mean linear intercept (L_M_). The same lungs and lobes were used across all the genotypes. For the aging study, both lungs were analyzed while for the cigarette smoke study, only the right lungs were analyzed. To calculate the L_M_, 5 randomly selected images of alveolar tissue per mouse were used. A 10 × 10 grid was superimposed over each image with ImageJ software (NIH, Bethesda, MD), and the number of alveolar intersections for each line was manually counted. L_M_ was calculated by dividing the length of lines by the number of intersections and averaged from 5 images for each mouse. Lines intersecting vasculature, bronchioles, or poorly inflated areas of the lung were not used. To determine whether *cftr* genotype contributes to the severity of cigarette smoke-induced emphysema, WT, Het, and KO mice were exposed to smoke from 3R4F research grade cigarettes (University of Kentucky, Lexington, KY) via whole-body exposure as previously described [18]. To model chronic tobacco smoking, the regimen consisted of 4 cigarettes per day, 5 days per week, for 10 months. Control mice of each genotype were exposed to filtered air instead of cigarette smoke. Four-nine mice were used for each group. Following the 10-month exposure, mice were euthanized. To investigate the possibility that CFTR genotype plays a role in the inflammatory response to cigarette smoke, another set of 8 mice from each genotype were subject to the same treatment regimen as above for 4 weeks. Bronchoalveolar lavage (BAL) was conducted by washing lungs twice with 2 ml of sterile phosphate-buffered saline (Life Technologies, Grand Island, NY). Cytospin was performed on BAL cells. Following Wright-Giemsa staining (Fischer Scientific, Kalamazoo, MI), numbers of macrophages/monocytes, neutrophils, and lymphocytes were counted. Studies were approved by the Ohio State University Institutional Animal Care and Use Committee (IACUC, protocol #2015A00000067), in accordance with NIH and OSU IACUC guidelines. Studies conducted at the University of Cincinnati were approved by IACUC protocol 06–04–07-01.

## Results

**Fig. 1:**
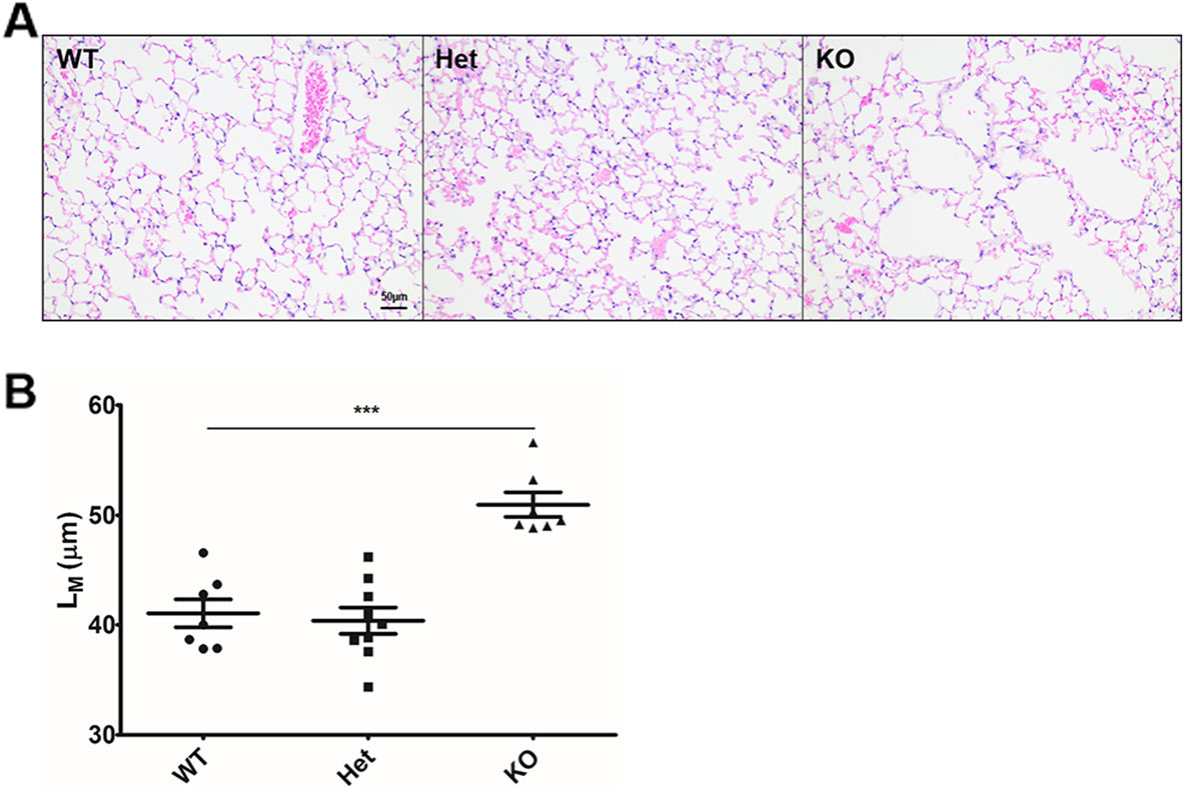
Development of emphysema-like changes in elderly mice. Healthy 14–20 month old wild-type (WT), CFTR-heterozygous (Het), and CFTR-knockout (KO) mice were sacrificed and lungs were fixed and inflated with formalin. (**a**) Representative micrographs of lung parenchyma stained with hematoxylin and eosin; and (**b**) mean linear intercepts (L_M_) of WT, Het, and KO mice. Magnification: 400X. Scale bar = 50 μm. ***p < .001 via one-way ANOVA with Tukey’s post-hoc multiple comparison. *N* = 7–9 mice per group

**Fig. 2:**
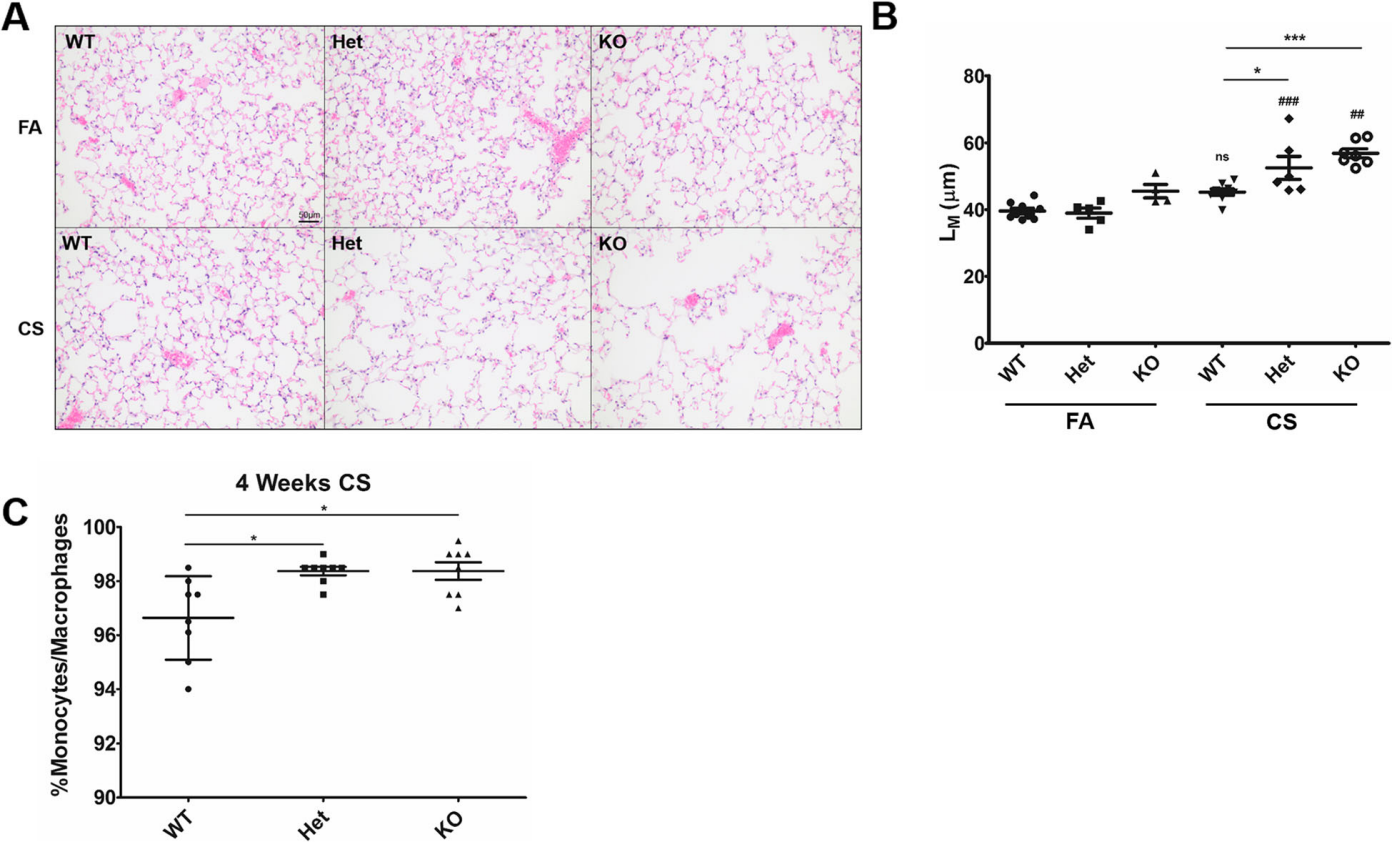
Emphysema-like changes in mice exposed to cigarette smoke. Mice wild-type (WT), CFTR-heterozygous (Het), and CFTR-knockout (KO) were exposed to filtered air (FA) or cigarette smoke (CS) for 10 months. (**a**) Representative micrographs of lung parenchyma stained with hematoxylin and eosin and (**b**) mean linear intercepts (L_M_) of wild-type (WT), CFTR-heterozygous (Het), and CFTR-knockout (KO) C57BL/6 J mice exposed to filtered air (FA) or cigarette smoke (CS) for 10 months. *N* = 4-9 mice per group. (**c**) Percentage of monocytes/macrophages in bronchoalveolar lavage fluid of mice exposed to cigarette smoke for 4 weeks. *N* = 8 from each group. Magnification: 400x. Scale bar = 50 μm. * *p* < .05, *** *p* < .001 between groups indicated by line; ## *p* < .01, ### *p* < .001 compared to FA mouse of same genotype. NS indicates there is no significant difference between FA and CS WT mice. Significance was determined via one-way ANOVA with Tukey’s post-hoc multiple comparison

Upon aging, Cftr-knockout mice displayed an increased mean linear intercept (L_M_), indicative of alveolar enlargement, compared to Heterozygous or WT mice (Figs. 1a &b). Among mice aged 14–20 months, there was no correlation between age and L_M_ (r^2^ = 0.1112). WT mice exposed to cigarette smoke (CS) for 10 months displayed a L_M_ that was increased but not significantly different from that of wild-type mice exposed to filtered air (FA). However, airspace enlargement in heterozygous and knockout mice exposed to smoke was significantly greater than their wild-type counterparts. Heterozygous and KO mice exposed to CS displayed increased L_M_ compared to their FA counterparts (Figs. 2a&b). Periodic Acid-Schiff staining was conducted on lung sections to determine mucus expression and obstruction, however very little mucus was detected in the lungs of all mice. No signs of infection were noted in the lungs of KO mice as evaluated by presence of the key inflammatory mediator IL-1β in BAL, presence of inflammatory cells, altered lung structure, or mouse weight loss. During the smoke exposure study, one WT mouse exposed to filtered air died from unknown causes (pathology was performed but the cause of death could not be identified). During the aging study, one heterozygous mouse had to be sacrificed due to edema and ocular swelling. In mice exposed to CS for 4 weeks, cytology revealed a modest but statistically significant increase in the percentage of macrophages/monocytes in BAL fluid between WT and both Het and KO mice (Fig. 2c). This increase in the percentage of BAL macrophages/monocytes was accompanied by a decrease in neutrophils (2.95 ± 1.30% for WT, 1.98 ± 0.67% for Het, and 1.97 ± 0.86% for KO mice), without a significant change in the total number of cells (7.74 ± 0.43 × 10^4^ for WT, 7.91 ± 0.42 × 10^4^, for Het, and 8.04 ± 0.42 × 10^4^ cells/mL for KO mice; *p* = 0.39 via ANOVA).

## Discussion

In the present study, we show using two different experimental models, aging and cigarette smoking, that lack of CFTR leads to increased airspace enlargement similar to emphysema. The finding of airspace enlargement in aged knockout mice is in accordance with findings in human CF patients [8]. However, we did not detect any mucus over-production or obstruction via periodic acid-Schiff staining. In fact, very little mucus was detected in the lungs of all mice (data not shown). We also did not detect any differences in inflammatory cell counts in the BAL fluid of mice at the time of sacrifice (data not shown), suggesting that complete absence of CFTR promotes emphysema upon aging independent of an external inflammatory or oxidative stimulus. Our finding that only KO aged mice develop emphysema, while both Het and KO mice exposed to cigarette smoke do, may suggest that decreasing CFTR expression sensitizes the lung to the effects of cigarette smoke. This may imply that individuals carrying CFTR mutations might be more prone to developing emphysema.

The mechanisms behind which CFTR dysfunction promotes emphysema are currently unclear. It has been suggested that CFTR dysfunction contributes to emphysema through its regulation of pro-inflammatory ceramide signaling [19]. In addition, it has been shown that CFTR inhibition increases the permeability of the pulmonary vasculature, and it has been hypothesized that this may lead to increased trafficking of inflammatory cells to the lung [20]. These studies however, were conducted using stressors such as cigarette smoke or lipopolysaccharide. Our novel finding in aged mice may suggest that an alternative, or at least an additional mechanism is playing a role.

The effect of aging on lung architecture has previously been investigated in BALB/c mice [21]. The authors found that while both alveolar surface area and volume increased, L_M_ did not change after 28 months [21]. L_M_ represents the alveolar volume to surface area ratio [22]. Thus, our results in WT and Het mice are in agreement with these findings [21]. Interestingly, several mouse models of accelerated aging also display signs of emphysema [23]. Mice homozygous for nonfunctional *klotho* have a maximal lifespan of about 12 weeks [24], and display alveolar epithelial cell apoptosis by 2 weeks of age, and increased L_M_ by 4 weeks [25]. Klotho is a Fibroblast Growth Factor-23 co-receptor with pleiotropic downstream signaling effects which are potentially related to its “Anti-aging” role, such as suppression of oxidative stress and senescence [26]. Another mouse model of accelerated aging is the Senescence Marker Protein-30 (SMP30) knockout mouse [27]. Compared to WT C57BL/6 controls, SMP30 KO mice displayed increased L_M_ beginning at 1 month and persisting up to 6 months of age [27]. In another study, SMP30 KO mice displayed increased L_M_ following 8 weeks of CS exposure, while WT mice did not [28]. Our finding that aged CFTR KO mice display increased L_M_ is in agreement with those in Klotho- and SMP30-KO mice, as well as several other strains of Senescence-Accelerated Mouse [23]. However, compared to the accelerated aging mice, CFTR KO mice take considerably longer (14 months) to display increased L_M_, and display normal lifespan due to gut correction (expression of human WT-CFTR in the intestinal epithelium under intestinal FABP promoter).

It is worth noting that similarly to the Klotho mouse, the CFTR KO mice display increased L_M_ later in life that does not further increase with age [25]. This suggests that our findings are not directly caused by a developmental defect associated with knocking out CFTR, which is important to note because it has been suggested that CFTR plays a role in lung development in both mice and humans [29]. It also suggests that our findings represent a discrete occurrence, rather than a continuous process. Thus, all mice in the range of 14–20 months were considered “aged” for this study. While many signs of cellular senescence have been noted in CF [30], further research will be necessary to determine if this explains our findings in aged CFTR KO mice. The main novelty of our findings in aged mice is that CFTR KOs develop increased L_M_, despite only poorly recapitulating the pathophysiology of human CF patients which is characterized by chronic lung infection [14, 15].

Considering the age-related nature of COPD, several studies have investigated whether aging itself increases susceptibility to the effects of cigarette smoke. One study noted that between 6 month- and 12 month-old mice, there were no differences in L_M_, recruitment of macrophages and neutrophils, or gene expression of several inflammatory cytokines in lung tissue following 8 weeks of CS exposure [31]. Another recent study found that following 6 months of CS exposure, there was no difference in L_M_ between young (14–16 months old) and old (23–24 months old) mice [32]. The study also found that aging enhanced the inflammatory response to CS, but did not exacerbate protein expression of the senescence marker p16, or expression of several senescence-associated genes. Interestingly however, aged mice not exposed to cigarette smoke did display an increased L_M_ and increased lung compliance, indicative of emphysema [32]. These results suggest that aging and CS contribute to COPD independently of each other, but not additively, even though age enhances CS-induced inflammation. Thus, our failure to detect differences in L_M_ any earlier than 10 months of CS exposure could more likely be explained by the requirement of time for the effects of CS to manifest, rather than the age of the mice. In the 4 week CS exposure study, we noted a change in BAL composition in both Het and KO mice compared to WT mice. This was characterized by a slight increase in macrophages/monocytes, and a slight decrease in neutrophils without affecting the total cell number. Whether slight changes in inflammatory profile are responsible for the change in lung architecture we report is difficult to ascertain but warrants further investigation.

In the present study, WT mice did not display increased L_M_ upon aging (between 14 and 20 months), however we did not age them past 20 months. Our finding that CFTR KO mice develop increased alveolar space enlargement similar to emphysema following aging or CS exposure may suggest a protective role for CFTR against both processes. This is further supported by our finding that CFTR Het mice develop emphysema only in response to CS. It is well established that CS decreases CFTR expression, while aging is not known to. In fact, a search for *cftr* in a recent transcriptomics database of the aging mouse lung shows that CFTR gene expression decreases by only 8% in alveolar type-II cells, and does not significantly change in any other cell type [33]. Further studies will be necessary to fully determine a mechanism for CFTR in protection against emphysema.

## Conclusion

To conclude, we report for the first time that mice lacking Cftr develop alveolar remodeling similar to emphysema upon aging, and that genetic reduction of Cftr expression contributes to emphysema-like changes following smoke exposure. Our results in aged mice may have implications for a CF population whose life expectancy is rapidly increasing [34]. Cystic fibrosis is associated with several age-associated pathologies, including diabetes and bone disease; and now emphysema. This may suggest emphysema as a significant future problem for aging CF patients, as well as foreshadow problems often associated with old age, such as neurological and cardiovascular diseases, which currently receive little basic research attention in the context of CF. Finally, CF carriership is fairly common [35], and our results in heterozygous mice exposed to cigarette smoke suggest that CF carriers may be more susceptible to the effects of cigarette smoke, including secondhand smoke, carrying significant cautionary implications. Our data may also suggest CFTR as an attractive therapeutic target in emphysema, however more research needs to be done.

## Data Availability

The analyzed datasets generated during the study are available from the corresponding author on reasonable request.

## Abbreviations

CF: Cystic Fibrosis;
CFTR: Cystic Fibrosis Transmembrane Conductance Regulator
COPD: Chronic Obstructive Pulmonary Disease
Het: Heterozygous (*cftr*^+/−^)
KO: Knockout (*cftr*^−/−^)
WT: Wild-Type
